# Supplementary material for: Excitonic insulator to superconductor phase transition in ultra-compressed helium
Source: Nat Commun. 2023 Jul 25;14:4458. doi: 10.1038/s41467-023-40240-x (PMC10368699; doi:10.1038/s41467-023-40240-x)
Supplement: Supplementary file 1 — Supplementary Information [file 41467_2023_40240_MOESM1_ESM.pdf]

Supplementary Information for "Excitonic Insulator to Superconductor Phase Transition in Ultra-Compressed Helium"

Cong Liu,[1] Ion Errea,[2] Chi Ding,[3] Chris Pickard,[4] Lewis J. Conway,[5] Bartomeu Monserrat,[6] Yue-Wen Fang,[7] Qing Lu,[3] Jian Sun,[3,*] Jordi Boronat,[1] and Claudio Cazorla[1,†]

[1]*Departament de Física, Universitat Politècnica de Catalunya, Campus Nord B4-B5, Barcelona 08034, Spain*
[2]*Fisika Aplikatua Saila, Gipuzkoako Ingeniaritza Eskola, University of the Basque Country (UPV/EHU), Europa Plaza 1, 20018 Donostia/San Sebastián, Spain; Centro de Física de Materiales (CSIC-UPV/EHU), Manuel de Lardizabal pasealekua 5, 20018 Donostia/San Sebastián, Spain; Donostia International Physics Center (DIPC), Manuel de Lardizabal pasealekua 4, 20018 Donostia/San Sebastián, Spain*
[3]*National Laboratory of Solid State Microstructures, School of Physics and Collaborative Innovation Center of Advanced Microstructures, Nanjing University, Nanjing 210093, China*
[4]*Department of Materials Science and Metallurgy, University of Cambridge, Cambridge CB30FS, United Kingdom; Advanced Institute for Material Research, Tohoku University, Sendai 980-8577, Japan*
[5]*Department of Materials Science and Metallurgy, University of Cambridge, Cambridge CB30FS, United Kingdom; Advanced Institute for Materials Research, Tohoku University, Sendai 980-8577, Japan*
[6]*Cavendish Laboratory, University of Cambridge, Cambridge CB30HE, United Kingdom.; Department of Materials Science and Metallurgy, University of Cambridge, Cambridge CB30FS, United Kingdom*
[7]*Fisika Aplikatua Saila, Gipuzkoako Ingeniaritza Eskola, University of the Basque Country (UPV/EHU), Europa Plaza 1, 20018 Donostia/San Sebastián, Spain; Centro de Física de Materiales (CSIC-UPV/EHU), Manuel de Lardizabal pasealekua 5, 20018 Donostia/San Sebastián, Spain*

[*] Corresponding author: jiansun@nju.edu.cn

[†] Corresponding author: claudio.cazorla@upc.edu

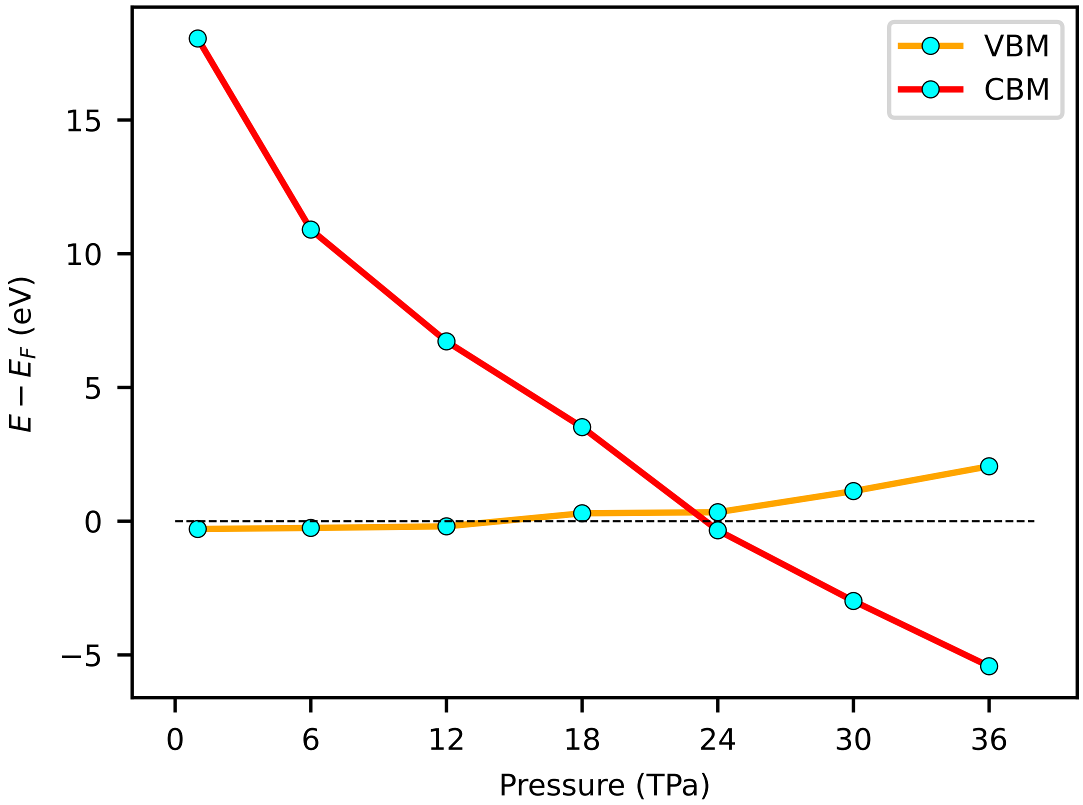

FIG. S1. Evolution of VBM and CBM levels in hcp ^{4}He expressed as a function of compression and calculated with the hybrid B3LYP functional. The dashed line represents the Fermi energy level. The crossing of the two curves indicates metallization. The CBM level (red curve), which presents full p-like electronic character, is efficiently tuned by pressure. The VBM level (orange curve), which presents mixed s–p like electronic character, hardly reacts to pressure below the predicted metallization point of 23 TPa.

TABLE S1. Cutoff energy convergence tests. Electronic density of states at the Fermi level, $N(E_F)$ (per formula unit), Fermi energy level, E_F, and EPC parameters calculated for the phonon at the wave vector Γ with branch index 4 and $q = (0.25, 0.53, 0)$ with branch index 1 considering the Monkhorst–Pack (MP) grid scheme and a Gaussian broadening of 0.002 Ry at 20 TPa.

q	Cutoff energy (Ry)	$N(E_F)$ (states/spin/Ry/formula)	E_F (eV)	$\lambda_{q\nu}$	$\gamma_{q\nu}$ (GHz)
(0,0,0)	100	0.011124	78.268714	39.9965	5191.84
	200	0.011294	78.267634	40.5615	5344.54
	300	0.011296	78.267626	40.5664	5345.93
	400	0.011295	78.267614	40.5634	5345.21
(0.25,0.43,0)	100	0.011124	78.268714	135.1200	8891.19
	200	0.011294	78.267634	140.5567	9375.37
	300	0.011296	78.267626	140.5894	9378.88
	400	0.011295	78.267614	140.5688	9377.09

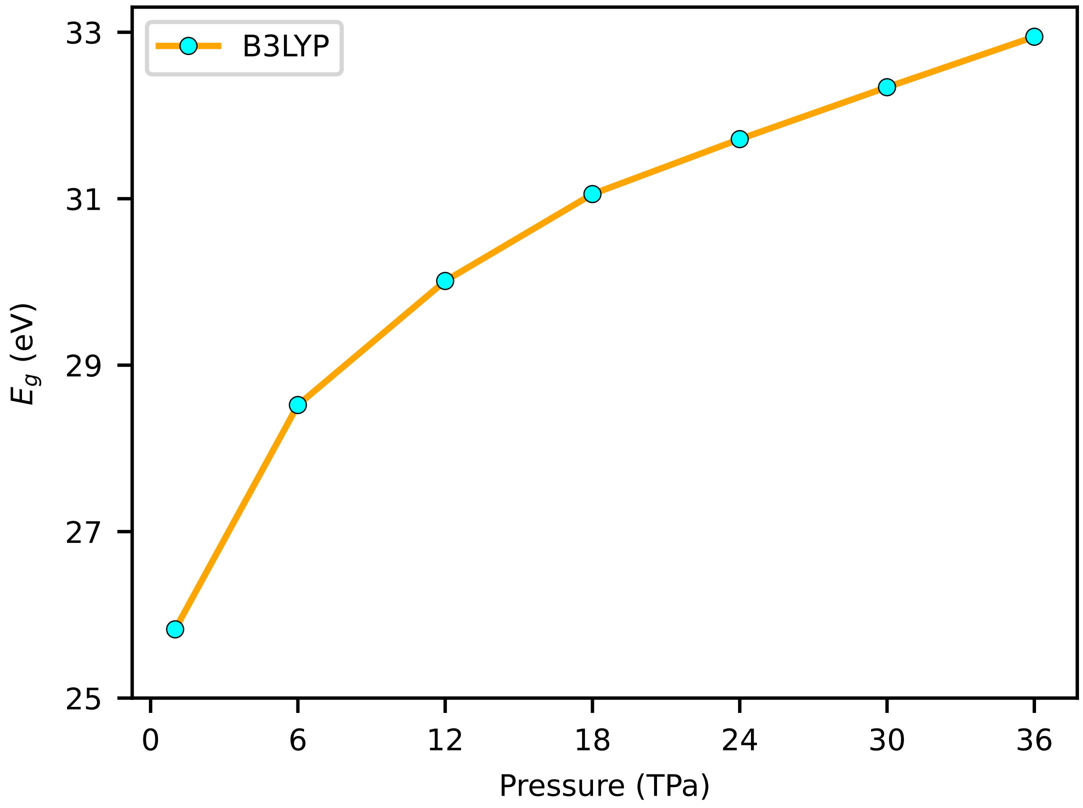

FIG. S2. Direct band gap at the reciprocal space point Λ point calculated in hcp ^{4}He as a function of pressure. The results were obtained with the hybrid B3LYP functional.

TABLE S2. Test on the **k**-mesh convergence for Γ and Λ points with a Gaussian broadening of 0.002 Ry at a pressure of 20 TPa.

**k**-mesh	λ_Γ	λ_Λ
96×96×48	35.9302	0.0011
112×112×56	35.1325	0.5302
128×128×64	38.2727	66.0029
144×144×72	33.4231	57.1476
160×160×80	40.1960	113.5229
176×176×88	39.1550	220.7670
192×192×96	40.5855	139.7354
256×256×128	44.5449	141.41328

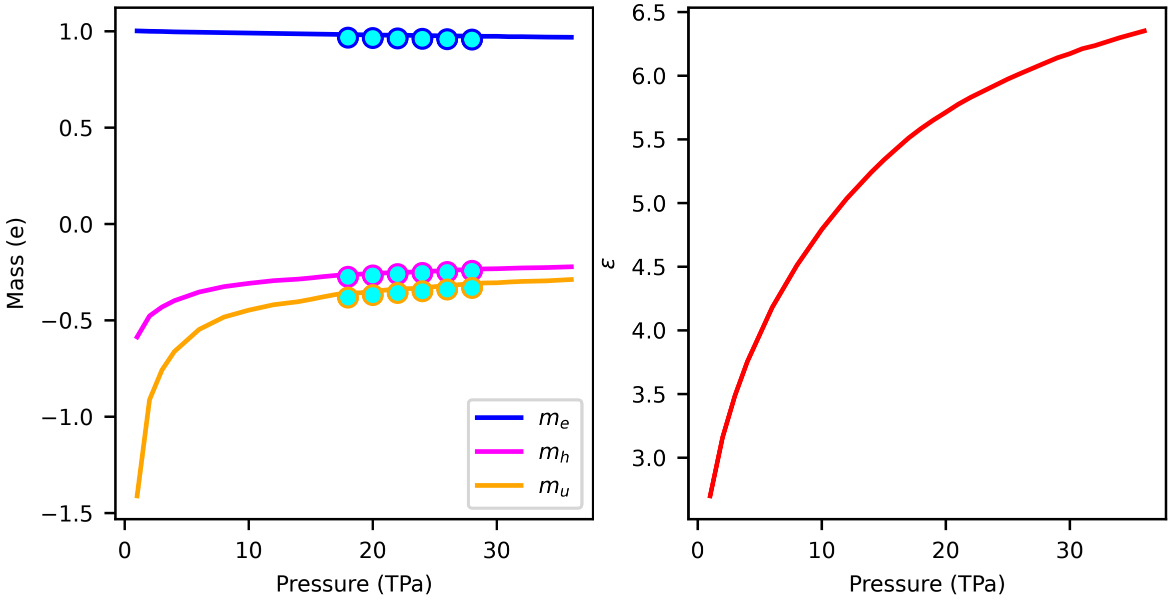

FIG. S3. Pressure evolution of the effective mass of electrons and holes and the relative dielectric constant in hcp ^{4}He. Solid curves are calculated with the semi-local PBEsol functional while the cyan points correspond to hybrid B3LYP calculations. It is found that the choice of the DFT functional does not appreciably affect the estimated effective masses of the electrons and holes as obtained from the curvature of the valence and conduction bands respectively. Thus, the choice of the DFT functional only influences noticeably the band gap.

TABLE S3. The EPC strength parameter λ, electronic density of states at the Fermi level $N(E_F)$ (per formula unit), logarithmic average phonon frequency ω_{log}, and critical superconducting temperature for metallic hcp ^{4}He at different pressures, P. The critical superconducting temperature was calculated with different approaches using $\mu^* = 0.10$ and 0.13 (the latter case within parentheses). T_c^{McM} was calculated with the McMillan formula, T_c^{AD} with the Allen-Dynes formula, and T_c^{mAD} with the modified Allen-Dynes formula.

P (TPa)	λ	ω_{log} (K)	$N(E_F)$	T_c^{McM} (K)	T_c^{AD} (K)	T_c^{mAD} (K)
20	0.45	3888	0.011294	30 (16)	31 (16)	32 (17)
30	0.22	4614	0.047406	2 (1)	2 (1)	2 (1)
40	0.38	4597	0.059410	16 (7)	16 (7)	17 (7)
50	0.52	5182	0.057635	71 (44)	73 (44)	76 (47)
100	0.52	6842	0.051989	97 (61)	99 (62)	103 (65)

TABLE S4. Band gap estimated with the DFT-PBE ($E_g^{\rm PBE}$), G_0W_0 ($E_g^{\rm G_0W_0}$), and GW ($E_g^{\rm GW}$) methods. The excitonic energy ($E_e^{\rm GW}$) and excitonic binding energy ($|E_{bind}|^{\rm GW}$) obtained with the many-body perturbation GW method at different pressures (P) are also shown.

P (TPa)	$E_g^{\rm PBE}$ (eV)	$E_g^{\rm G_0W_0}$ (eV)	$E_g^{\rm GW}$ (eV)	$E_e^{\rm GW}$ (eV)	$\|E_{bind}\|^{\rm GW}$ (eV)
22.25	-3.81314	-1.24285	0.86015	–	–
22.5	-3.98497	-1.4352	0.66742	–	–
22.75	-4.14673	-1.62281	0.53998	0.19071	0.34927
23	-4.3114	-1.82339	0.35686	0.01275	0.34411
23.25	-4.49332	-2.01736	0.15883	-0.17623	0.33623

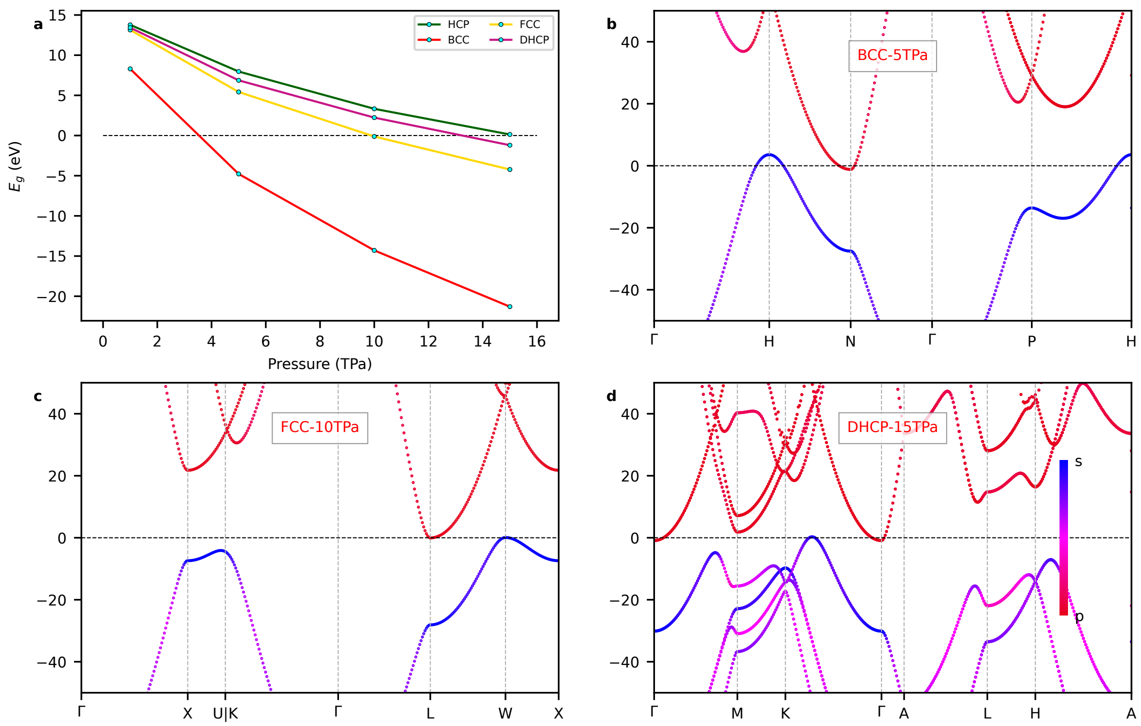

FIG. S4. Electronic band-structure properties calculated for ^{4}He considering other possible crystal structures, namely, body-centered cubic (bcc), face-centered cubic (fcc), and double hexagonal closed packed (dhcp). **a** Band gap expressed as a function of pressure and calculated with the semi-local PBEsol functional. **b-d** Projected electronic band structure of metallic helium calculated in the bcc, fcc and dhcp phases, respectively.

TABLE S5. Test on the **q**-mesh convergence for ω_{log} and T_c with Coulomb pseudopotential $\mu^* = 0.10$ at a pressure of 50 TPa.

**q**-mesh	λ	ω_{log} (K)	T_c (K)
6×6×3	1.45	6300	695
8×8×4	1.75	5047	658
12×12×6	0.33	5772	7
14×14×7	0.39	5441	21
16×16×8	0.52	5203	72

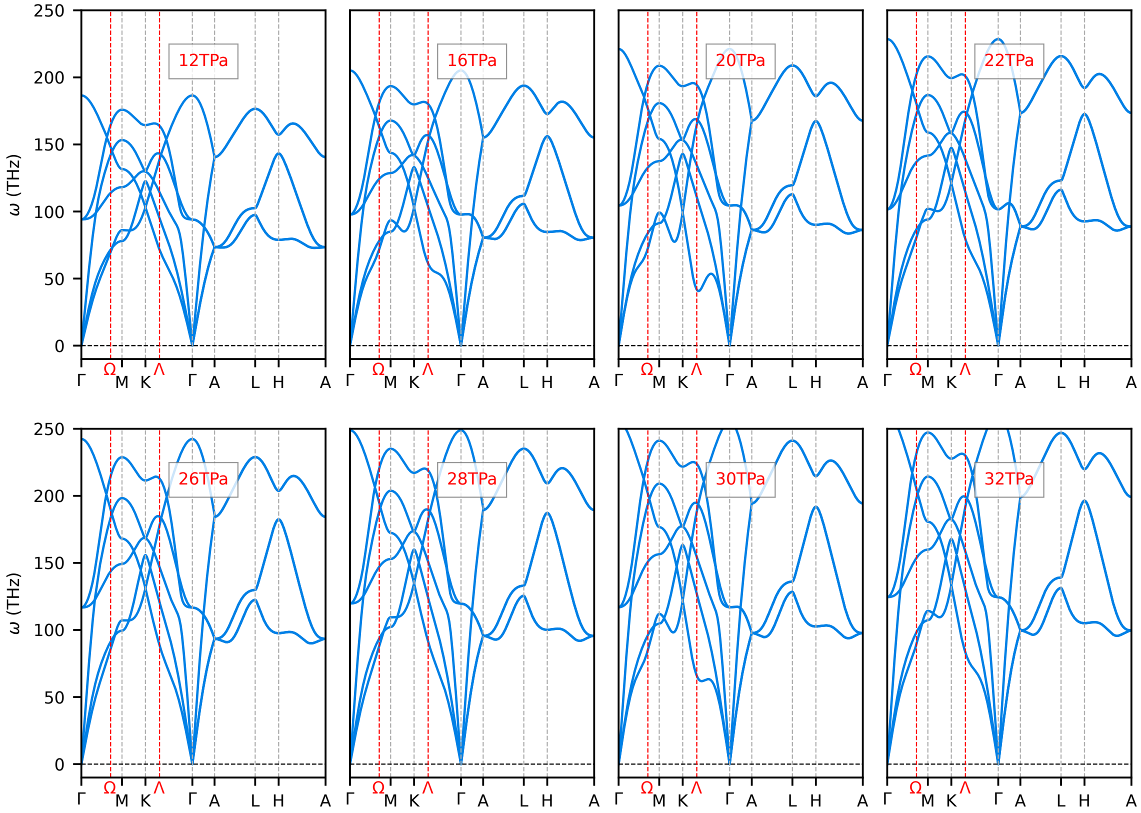

FIG. S5. The phonon spectrum of hcp ^{4}He calculated at different pressures using the semi-local PBEsol functional in VASP. The Λ and Ω reciprocal space points, which are not a highly symmetric, correspond to the VBM and the secondary VBM levels, respectively.

TABLE S6. Comparison of the band gap, E_g (eV), and excitonic binding energy, $|E_{bind}|$ (eV), obtained for hcp ^{4}He disregarding and considering dynamical electron-phonon coupling (EPC) effects. EPC effects were taken into consideration by generating 10 distorted supercells in which the atoms where displaced according to representative thermal lines (see main text).

configuration	$\|E_{bind}\|$	E_g
1	0.256	1.315
2	0.215	1.408
3	0.213	1.266
4	0.253	1.475
5	0.299	1.192
6	0.232	1.157
7	0.258	1.220
8	0.420	1.407
9	0.384	1.482
10	0.183	1.511
dynamical (with EPC effects)	0.271	1.343
static (without EPC effects)	0.192	0.316

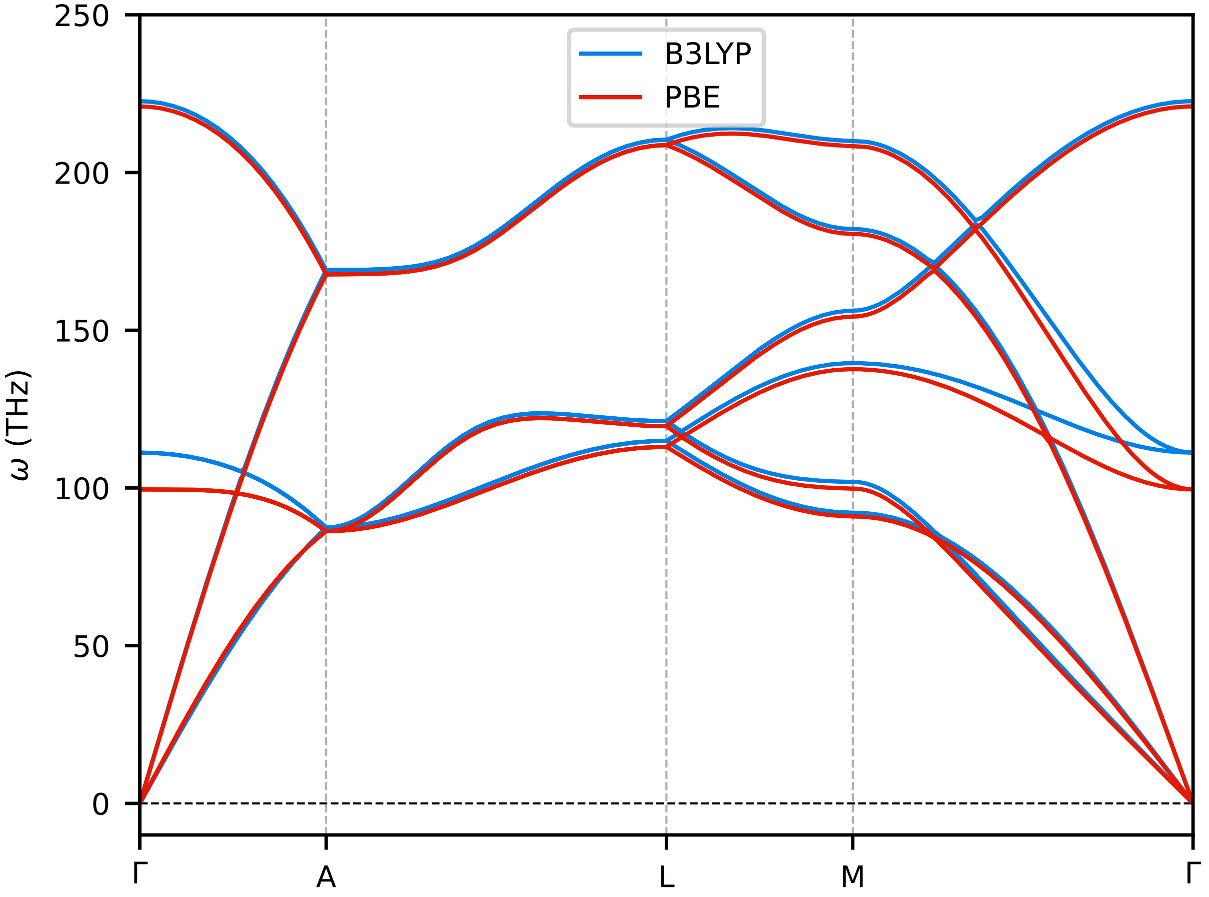

FIG. S6. Comparison of the phonon spectrum calculated with the semi-local PBE and hybrid B3LYP functionals for a relatively small $2 \times 2 \times 2$ supercell.

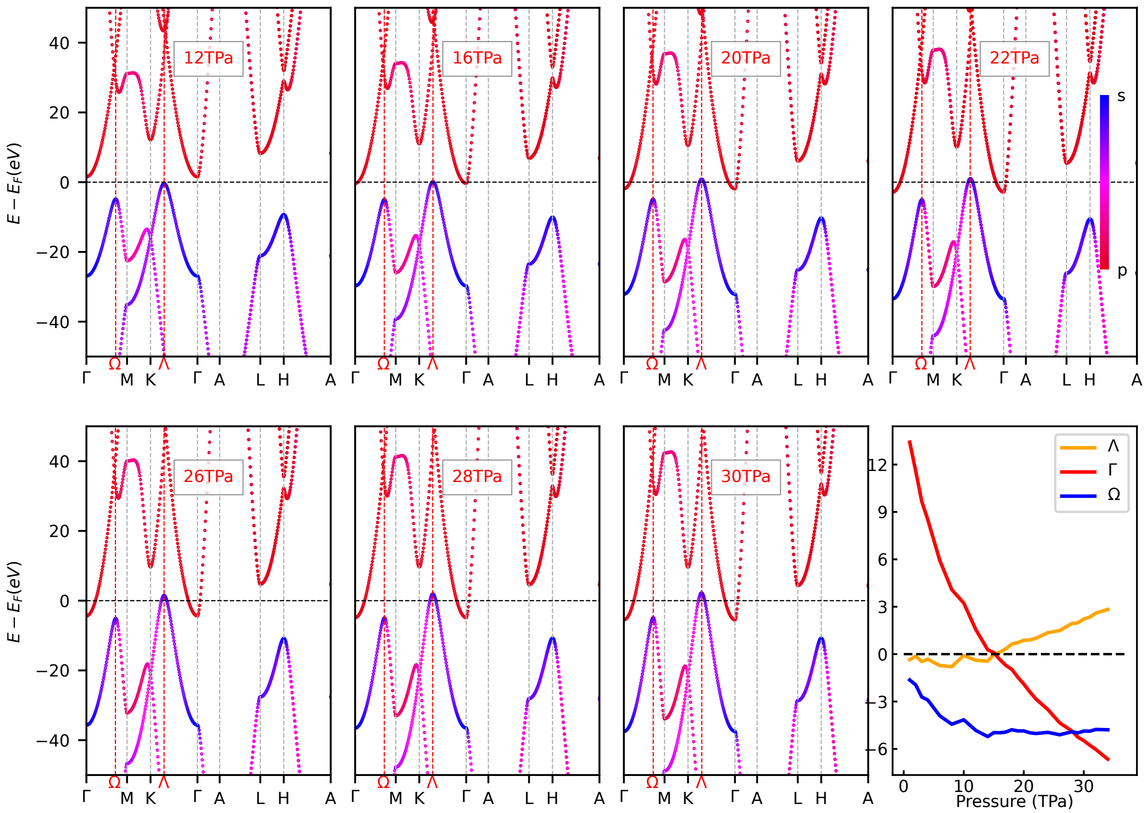

FIG. S7. Projected electronic band structure of hcp ^{4}He at different pressures calculated with the semi-local PBEsol functional. The Λ and Ω reciprocal space points, which are not highly symmetric, correspond to the VBM and secondary VBM levels, respectively. A red-magenta-blue color scale is used to represent the dominant character of the valence and conduction bands; red denotes s-like character and blue p-like. The last panel shows the pressure-induced energy evolution of the relevant reciprocal space points Λ, Γ and Ω point, as referred to the Fermi surface.

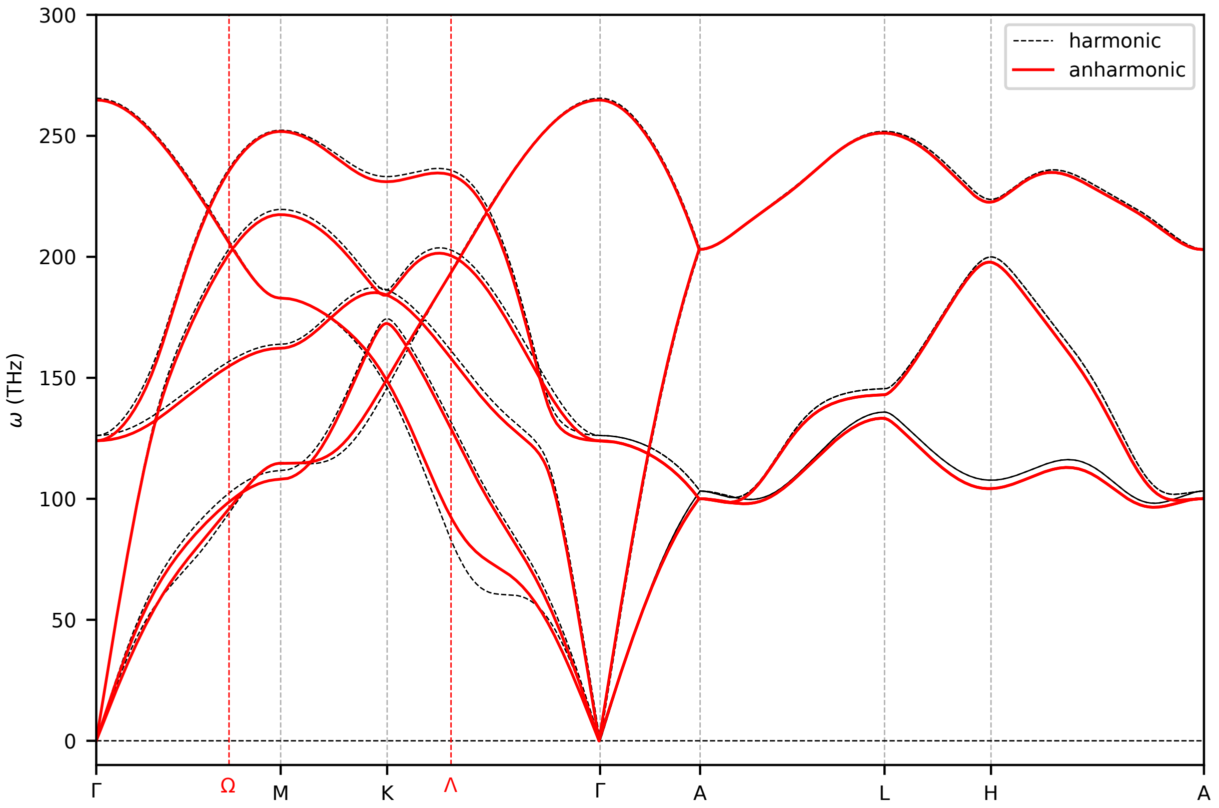

FIG. S8. Quantum anharmonic effects on the phonon spectrum of ultra-compressed hcp ^{4}He. The comparison between the harmonic (dashed black lines) and the anharmonic (solid red lines) phonon spectra. The harmonic phonon spectra are taken from the DFPT calculations and the anharmonic phonon spectra are calculated from the free energy Hessian dynamical matrix $\mathcal{D}^F$ up to the third-order force constants.

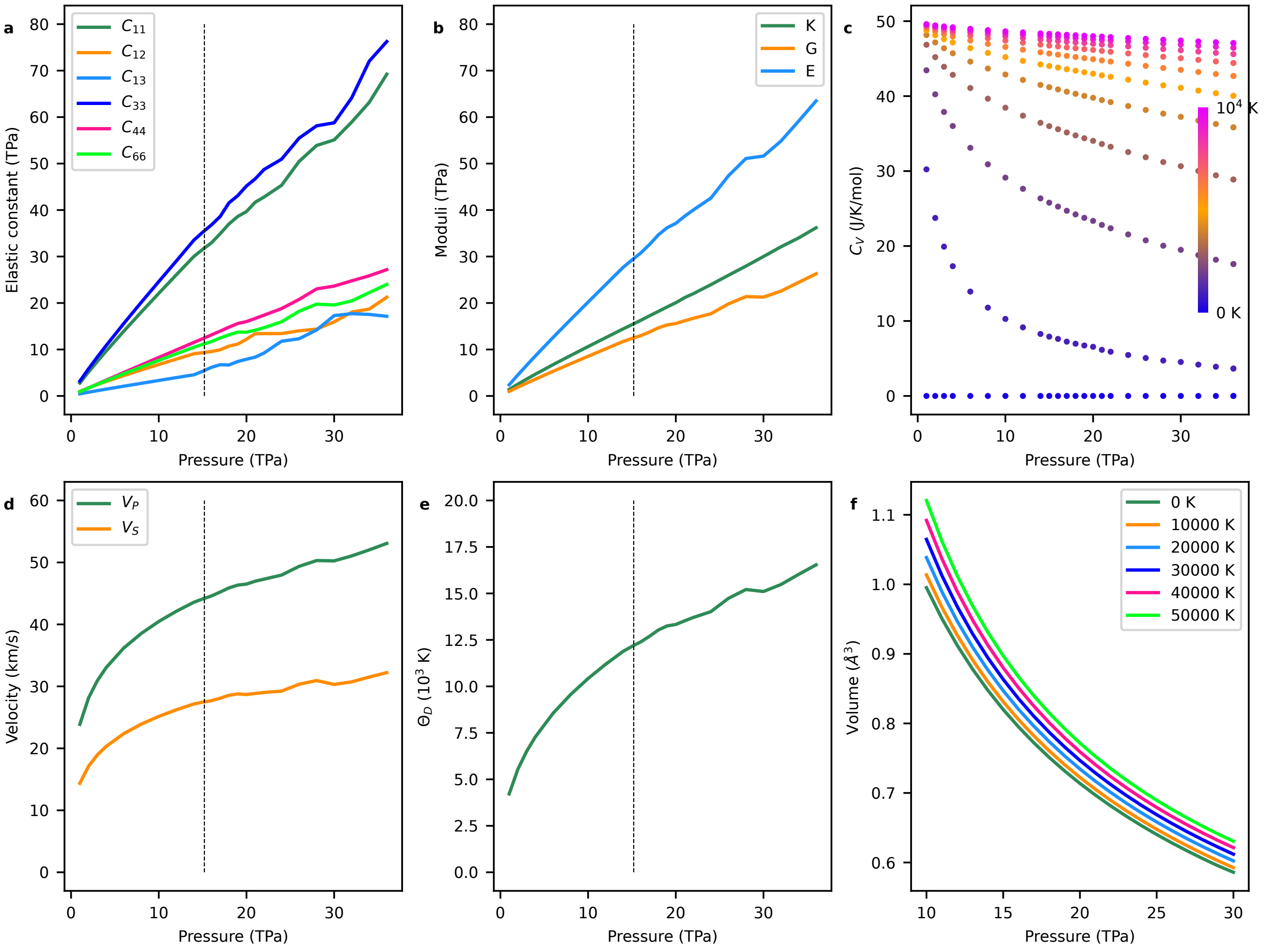

FIG. S9. Elastic properties of hcp ^{4}He expressed as a function of pressure and calculated with the semi-local PBEsol functional. **a** Elastic constants $\{C_{ij}\}$. **b** Bulk modulus (K), shear modulus (G), and Young's modulus (E). **c** The heat capacity (C_V) of the crystal calculated at fixed volume V. C_V was calculated within the temperature interval $0 \leq T \leq 10,000$ K. **d** Elastic sound wave velocity: longitudinal wave velocity (v_p) and transverse wave velocity (v_s). **e** Debye temperature (Θ_D) estimated for metallic helium. **f** Temperature-dependence of the equation of state of hcp ^{4}He as estimated with the quasi-harmonic approximation. The vertical dashed line in the figures marks the metallization pressure (i.e., 15 TPa as obtained with the semi-local PBEsol functional). In the insulating phase, the pressure-dependence of the elastic constants and moduli are almost linear. In the metallic state, lattice distortions occur and those curves depart from the pressure-dependent linear behaviour; there is also a crossing between the C_{12} and C_{13} lines.

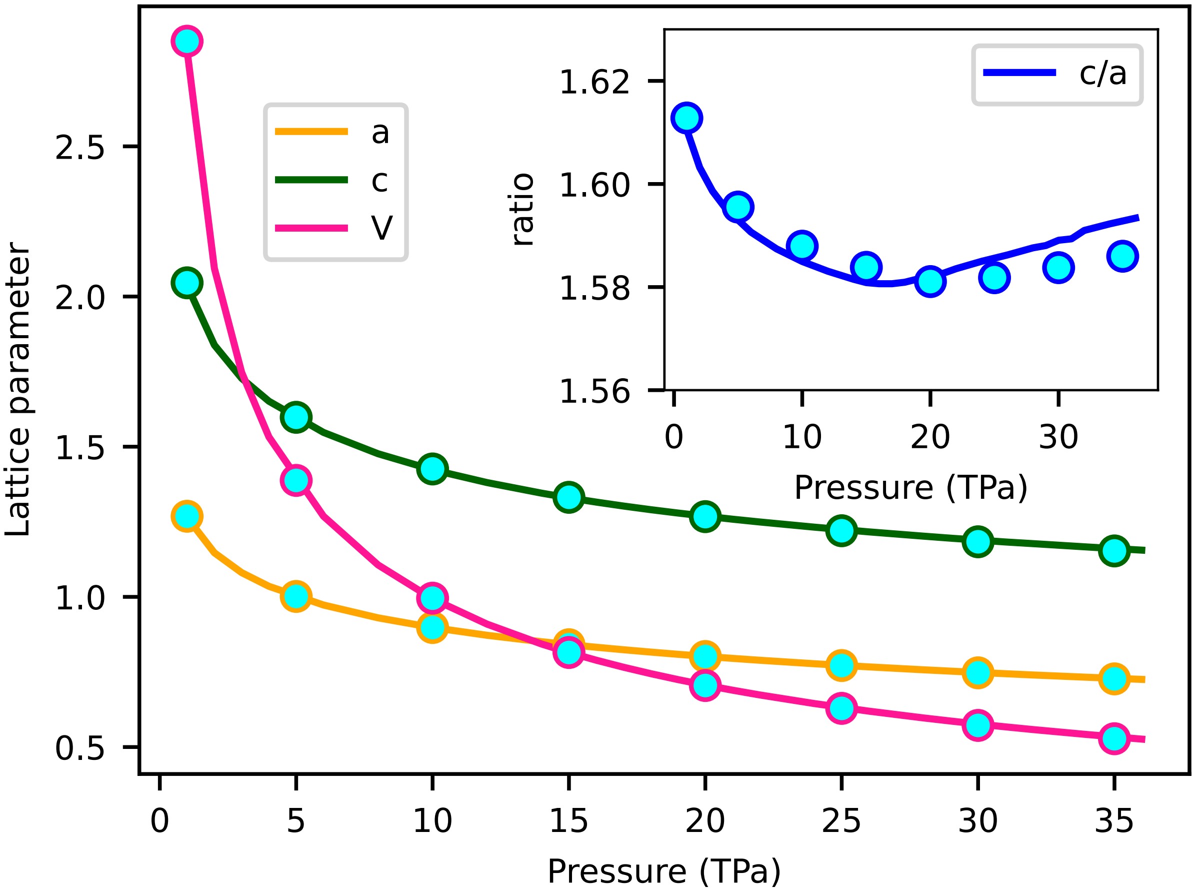

FIG. S10. Comparison of the equation of state of hcp ^{4}He obtained with two different DFT software: Quantum Expresso (QE, circles) and VASP (curves). The length of the a and b lattice parameters (Å) and total volume (Å^3) for unit cell with 2 atoms expressed as a function of pressure and calculated with the QE and VASP codes agree remarkably well. The agreement between the two DFT codes on the value of the c/a ratio, however, is not exact within the pressure range $20 \le P \le 30$ TPa. VASP calculations were performed with projector augmented-wave pseudopotentials while QE calculations were performed with ultrasoft pseudopotentials.

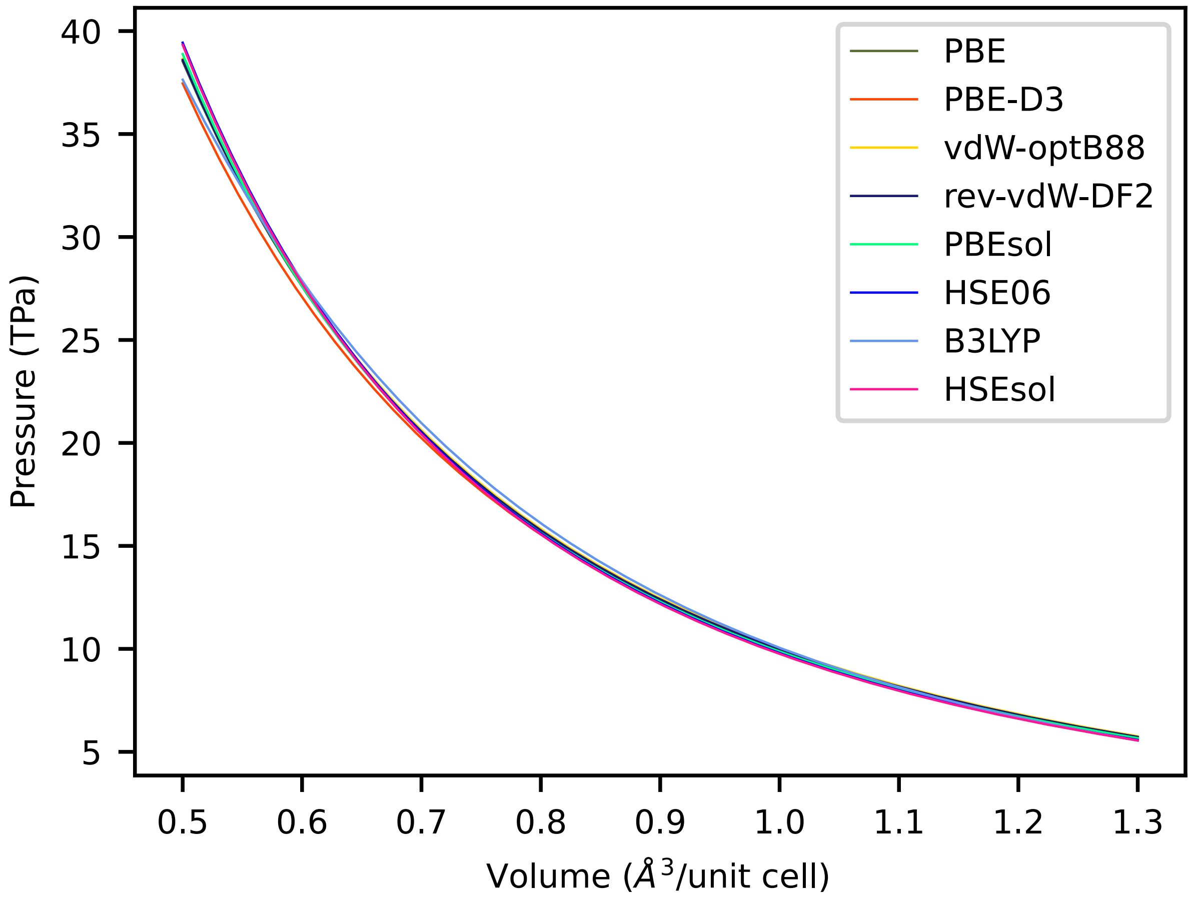

FIG. S11. Comparison of the equation of state of hcp ^{4}He obtained with different functionals.

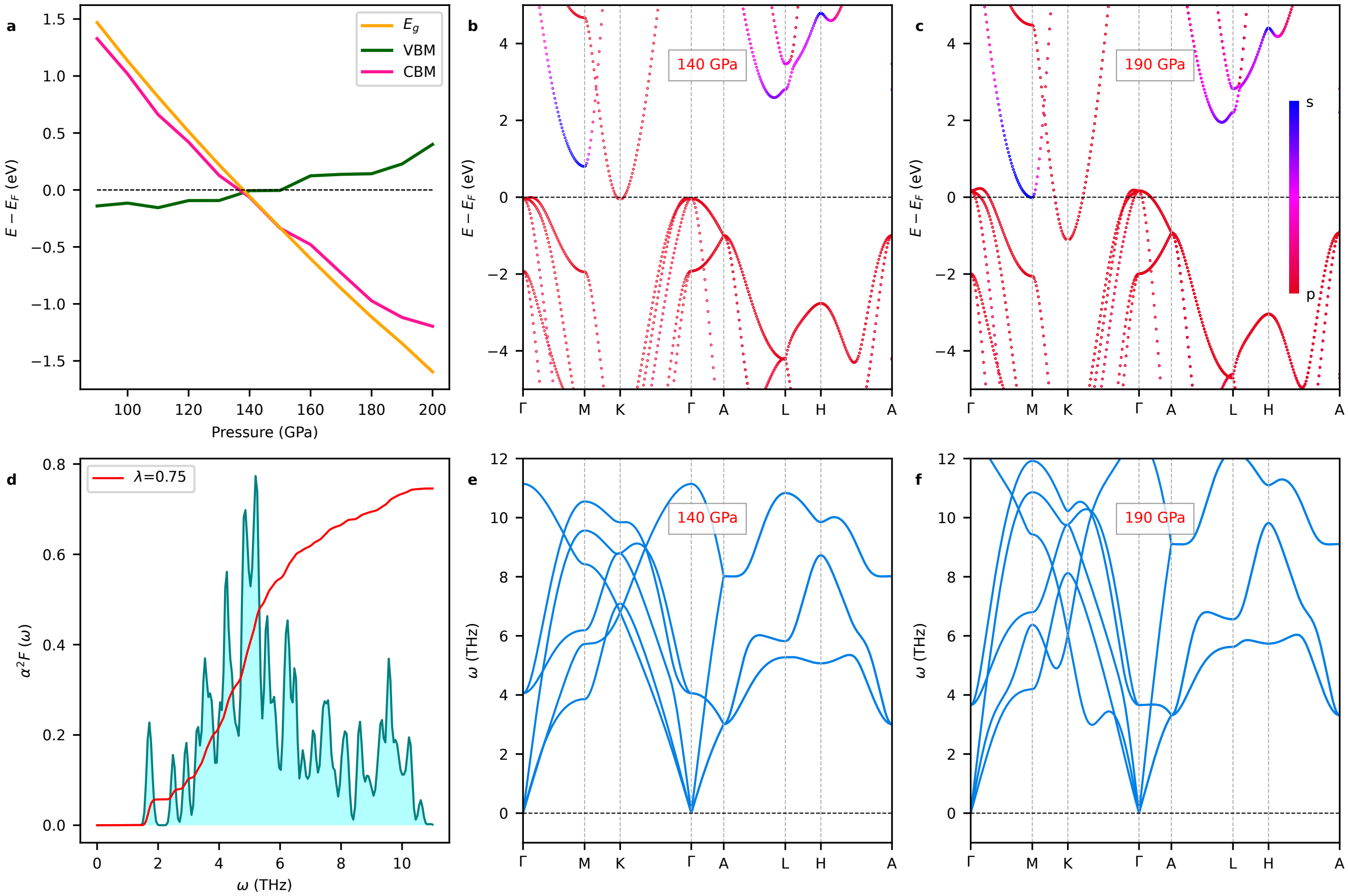

FIG. S12. Excitonic insulator state and superconductivity in solid hcp xenon. **a** Band gap, VBM and CBM pressure-induced evolution. **b-c** Projected electronic band structure and **e-f** Phonon spectrum of hcp Xe at 0.14 and 0.19 TPa calculated with the semi-local PBEsol functional. **d** Eliashberg spectral function, $\alpha^2F(\omega)$, estimated at 0.14 TPa along with the integrated electron–phonon coupling constant λ. In analogy to helium, solid xenon also maintains the hcp crystal structure at high pressures (i.e., $\sim$ 0.1 TPa). For the electronic band structure and phonon spectrum calculations, we considered the electrons $5s^25p^6$ as valence states and used the semi-local PBEsol DFT functional with a plane wave cutoff of 300 eV. For EPC calculations, we used ultrasoft pseudopotentials, a plane-wave cut-off energy of 50 Ry for the kinetic energy and of 500 Ry for the charge density. We adopted a dense and shifted k-point mesh of $16 \times 16 \times 8$ for the self-consistent calculation and then a denser $32 \times 32 \times 16$ grid for further EPC calculations. As it is shown the figure, solid xenon becomes metallic at an experimentally accessible pressure of 0.14 TPa. Its band gap is also indirect with the VBM located at the Γ point and the CBM at K point. VBM and CBM exhibit pure electronic p-like character while the secondary CBM s-like. No phonon softening occurs at 0.14 TPa as the band gap closure involves p-p orbital interactions. Meanwhile, phonon softening is observed at 0.19 TPa upon closure of the secondary band gap which involves s-p orbital interactions, in analogy to solid helium. The Eliashberg spectral function calculated at 0.14 TPa shows that the electron phonon coupling strength (λ = 0.75) mainly stems from the low-frequency region (i.e., 2–6 THz). In this case, the resulting critical superconducting temperature is 10 K.

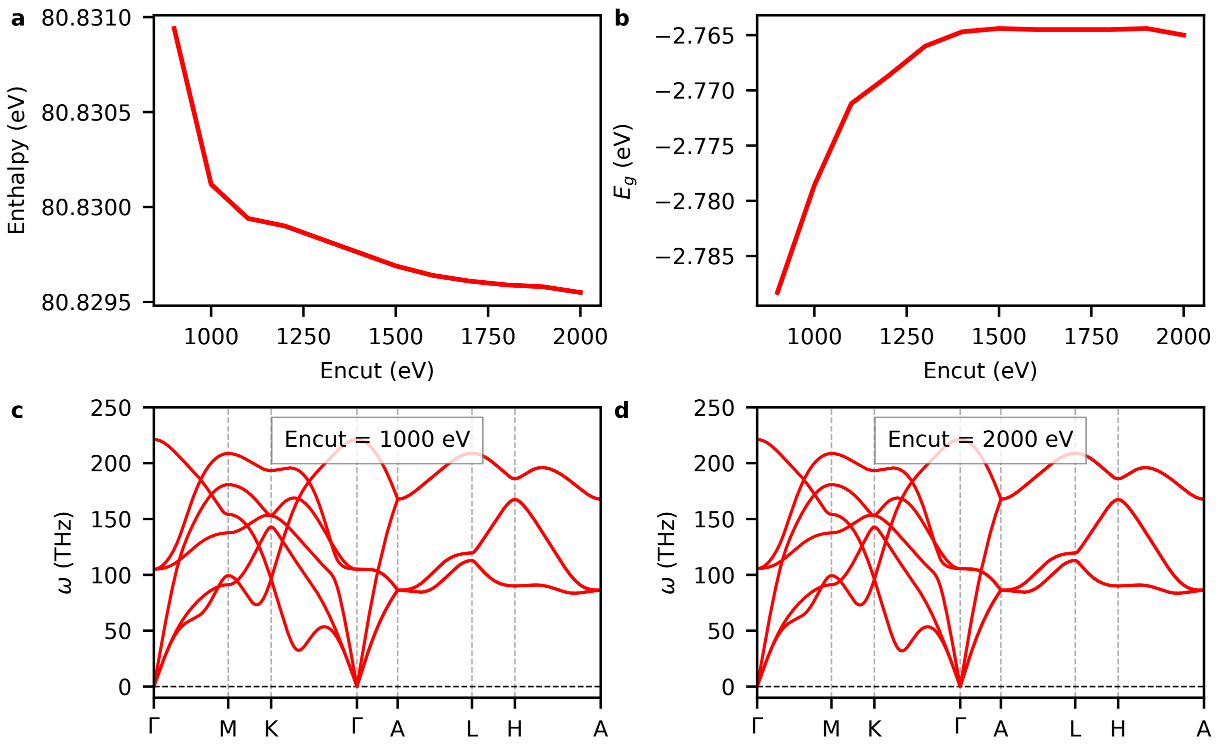

FIG. S13. Energy cutoff tests performed with the VASP code and the semi-local PBEsol functional. Results are shown for the **a** enthalpy, **b** band gap and **c-d** phonon spectrum of solid helium at 20 TPa. It is found that an energy cutoff of 1500 eV guarantees convergence in all the quantities to the desired level of accuracy.

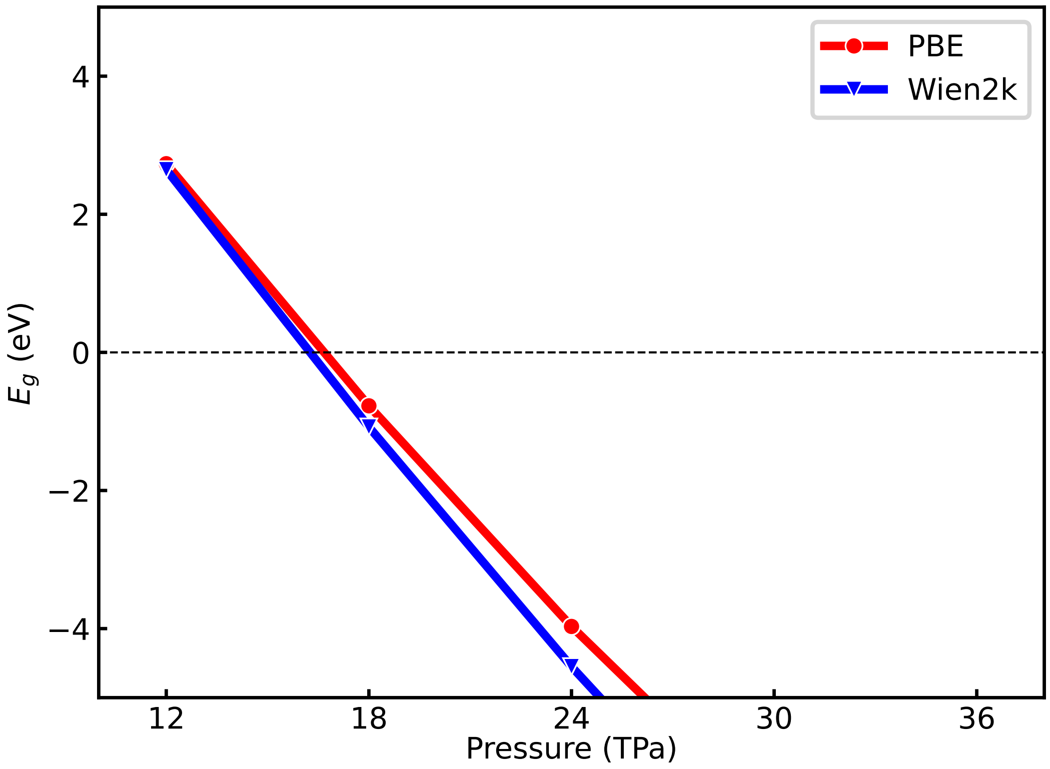

FIG. S14. Comparison of the PBE band gap evolution obtained with a PAW pseudopotential (red line, results obtained with the VASP code) and an all-electron approach (blue line, results obtained with the WIEN2K code). The full-potential (linearized) augmented plane-wave method is consistent with the employed pseudopotential PBE approach when evaluating band structure closure under such a high pressure.

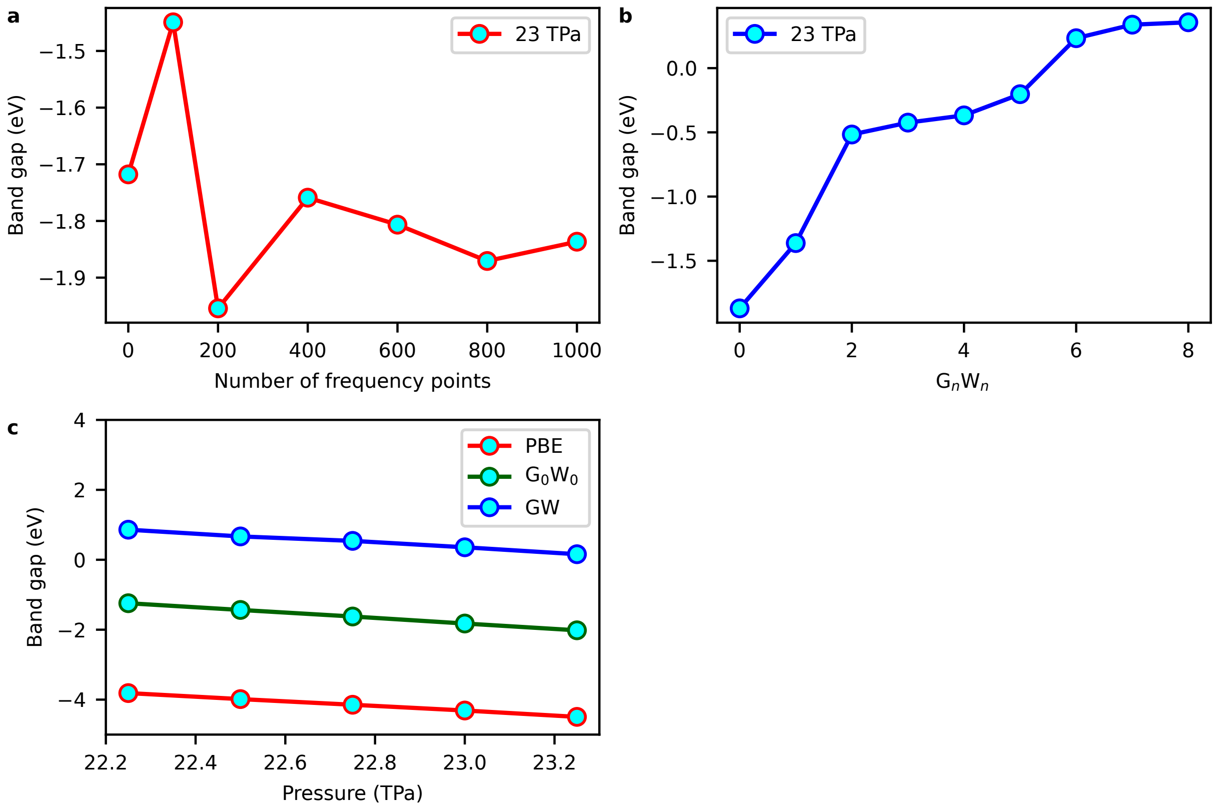

FIG. S15. Convergence of the calculated GW band gap with respect to **a** the number of frequency points and **b** number of iterations on G_0W_0. **c** The band gap estimated for five different pressures and obtained from PBE, G_0W_0, and full GW calculations.

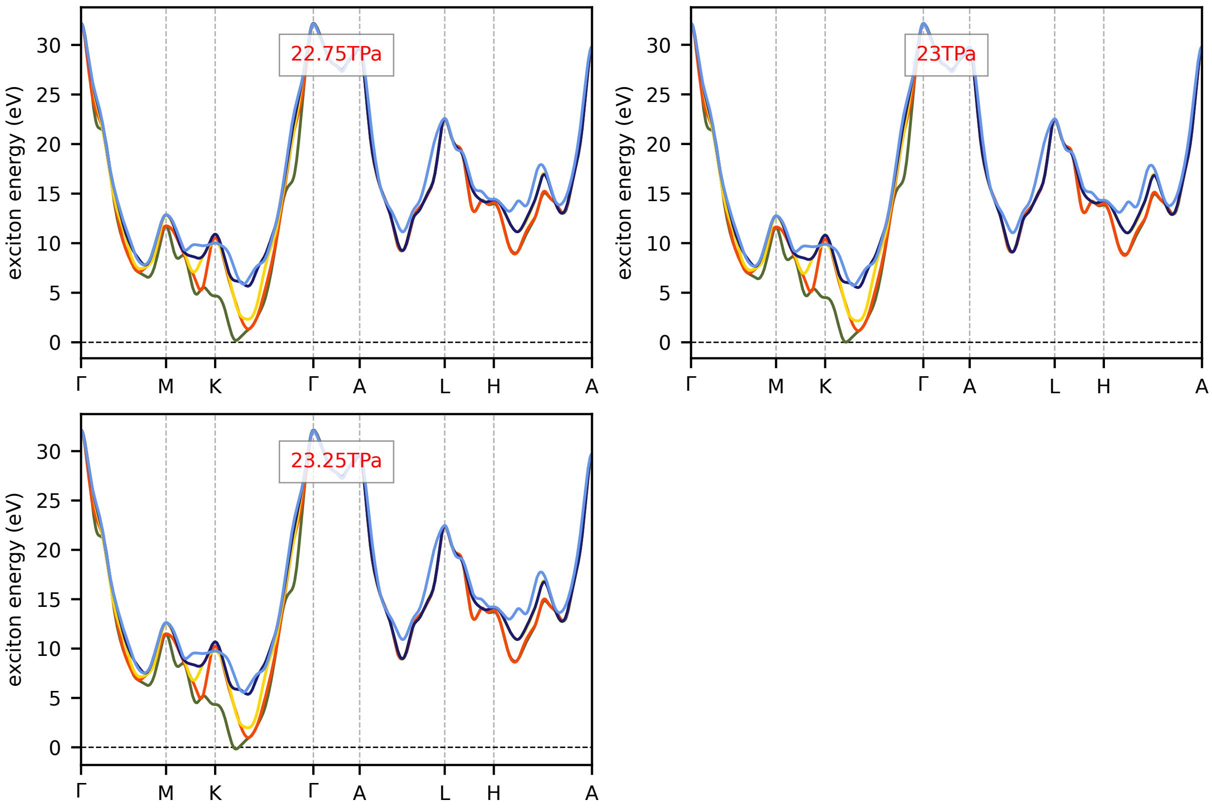

FIG. S16. Pressure evolution of the excitonic energy obtained with many-body perturbation GW calculations. The interpolated exciton energies are mapped into different **q**-point with colorful curves indicating different bands. The exciton in path K–Γ has the lowest energy with a value of −0.34 eV at 23.25 TPa, which is negative, indicating that the exciton state is bound.

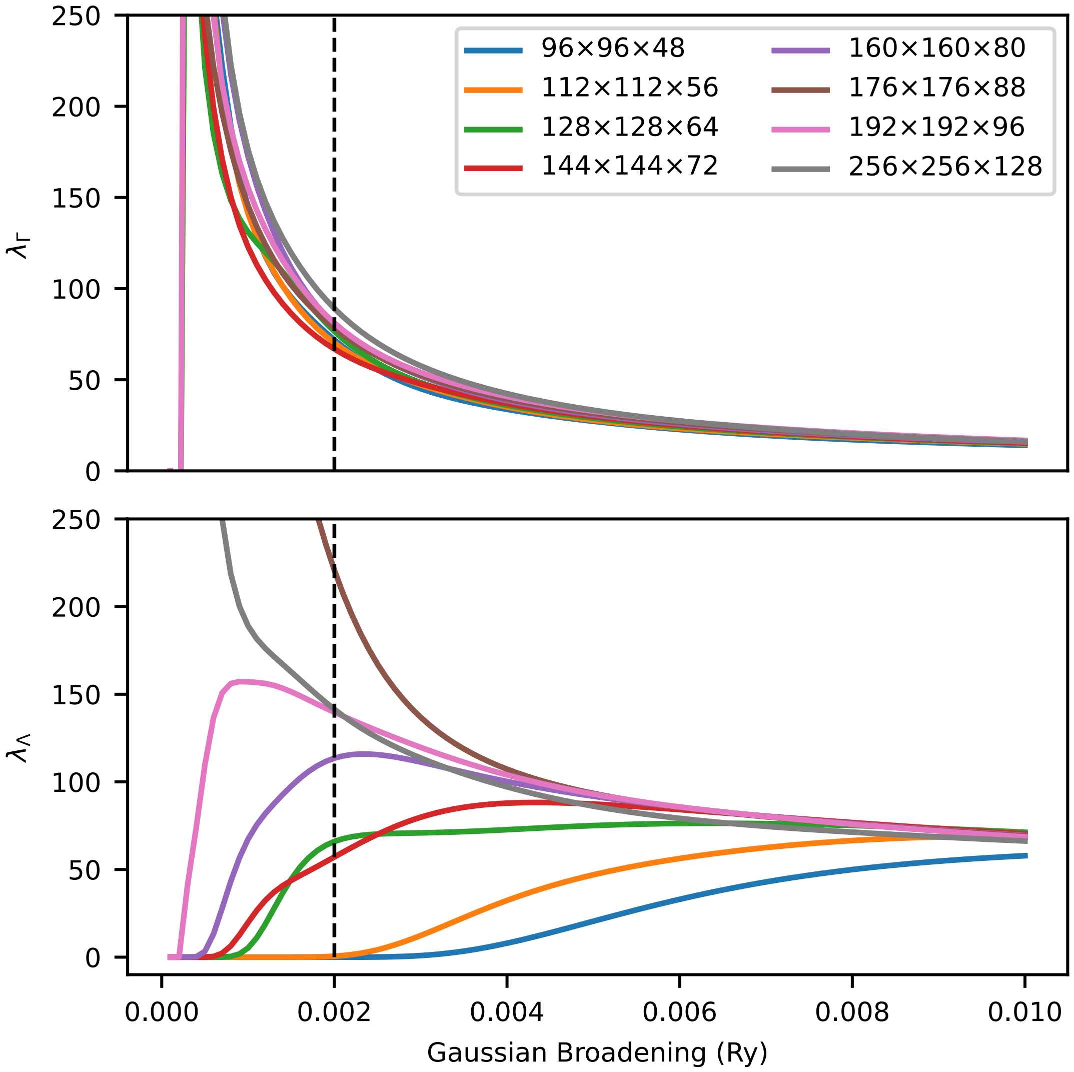

FIG. S17. **k**-mesh convergence test for the electron phonon coupling strength at the Γ and Λ points and a pressure of 20 TPa. It is shown that the convergence of λ_Γ with respect to **k**-mesh is most consistent. We chose a **k**-mesh of 192×192×96 with a Gaussian broadening of 0.002 Ry, which guarantees that λ is properly converged as compared to the result obtained with the largest **k**-mesh of 256×256×128.

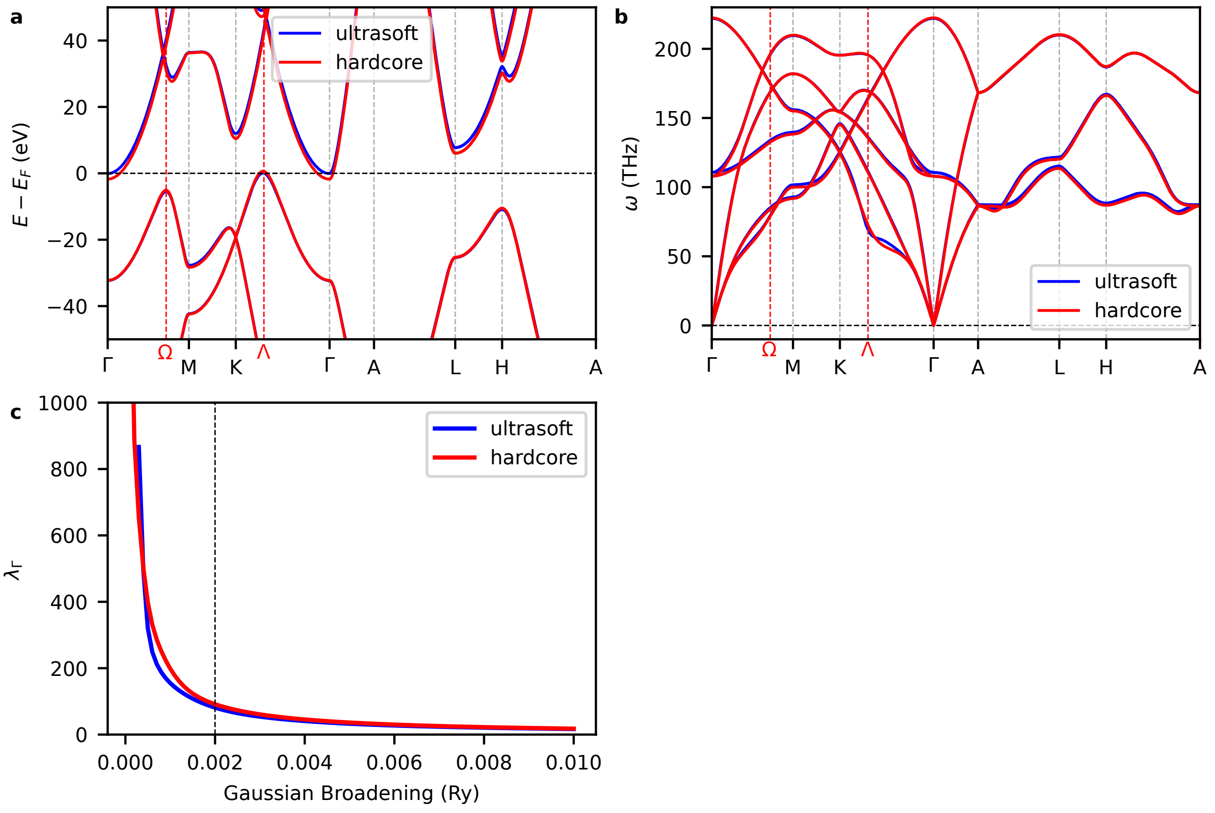

FIG. S18. Test on the pseudopotentials. We compare **a** bandgap structure, **b** phonon spectrum, **c** electron phonon coupling strength at the Γ point and 20 TPa. It is shown that ultrasoft pseudopotentials are reliable for conducting calculations in the TPa regime.

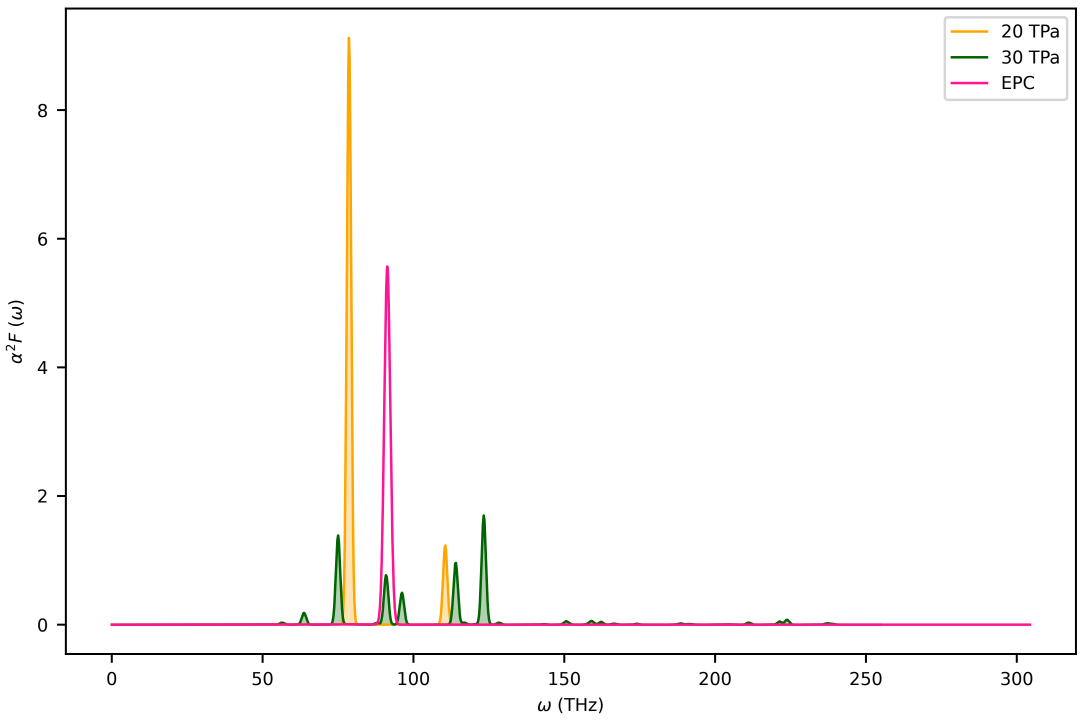

FIG. S19. Comparison of the Eliashberg spectral function obtained at 20 and 30 TPa by disregarding EPC effects and at 30 TPa by considering electron-phonon coupling effects on the band gap (EPC).
